# Idiopathic Aortitis With Retroperitoneal Fibrosis Course and Its Treatment

**DOI:** 10.7759/cureus.17366

**Published:** 2021-08-22

**Authors:** Zaheer Faizi, Ammar Humayun, Marissa Matto, Zeyn White, Sai Sajja

**Affiliations:** 1 General Surgery, Crozer-Chester Medical Center, Upland, USA; 2 Surgery, Crozer-Chester Medical Center, Upland, USA; 3 Surgery, Drexel University College of Medicine, Philadelphia, USA; 4 Surgery, Trinity School of Medicine, Kingstown, VCT; 5 Vascular Surgery, Crozer-Chester Medical Center, Upland, USA

**Keywords:** aortitis, retroperitoneal fibrosis, idiopathic aortitis, idiopathic, rheumatoid vasculitis, systemic lupus erythematous disease

## Abstract

Aortitis is an inflammatory phenomenon involving one or more layers of the aorta and can have infectious or noninfectious etiologies. Complications of aortitis include aneurysm, dissection, and rupture, which can lead to ischemic organs and ultimately death. Noninfectious aortitis is often secondary to trauma or results from a systemic inflammatory process. It is further categorized based on clinical characteristics, laboratory findings, and imaging. There are some cases in which the etiology cannot be determined and is, therefore, idiopathic in nature. We present a case of a 67-year-old male who presented with malaise, abdominal pain, anorexia, and significant weight loss for several months. Imaging revealed retroperitoneal fibrosis and aortitis. After an extensive workup, we diagnosed idiopathic aortitis and treated the patient with high-dose corticosteroids that led to symptom improvement.

## Introduction

Aortitis is defined as the inflammation of any of the layers of the aorta. Longstanding untreated or undiagnosed aortitis can lead to aneurysm, dissection, and eventually rupture. Other complications include ischemia of organs, compression, and death. Aortitis can be divided into two broad types: infectious and noninfectious. Infectious aortitis results from the insult of the aortic wall by bacterial, mycobacterial, fungal, and viral pathogens. Noninfectious aortitis is often caused by trauma or an inflammatory process. Further delineation on type is based on clinical characteristics, laboratory findings, and/or imaging. Noninfectious aortitis, associated periaortitis, and retroperitoneal fibrosis are often idiopathic without any clear inciting event or cause. We present a case of a 67-year-old male who experienced malaise, loss of appetite, abdominal pain, and considerable weight loss for several months. Imaging revealed retroperitoneal fibrosis along with aortitis. After extensive workup, we diagnosed idiopathic aortitis. He was subsequently treated with high-dose steroids and his symptoms subsequently improved.

## Case presentation

A 67-year-old male with a past medical history of hypothyroidism and gastroesophageal reflux disease (GERD) visited his primary care doctor with a five-week history of malaise, low energy, and abdominal discomfort associated with nausea after every meal. On initial presentation, the patient localized the pain to his mid-abdomen. Ten days prior to the presentation, he had experienced four days of low-grade fever. The patient endorsed a 14.5-kg (32-lb) weight loss since the onset of symptoms. His initial WBC count was 7.6 thousand/uL (normal range: 3.8-10.8 thousand/uL) and platelets were slightly elevated at 408 K/uL (normal range: 140-400 K/uL). His initial basic metabolic panel was unremarkable. Notably, his ferritin level was elevated at 1403 ng/mL (normal range: 24-380 ng/mL). The hepatic panel was within normal limits. Erythrocyte sedimentation rate (ESR) was elevated at 47 mm/h (normal range: 0-30 mm/h), and c-reactive protein (CRP) was elevated at 22 mg/L (normal range: 0-10 mg/L).

**Figure 1:**
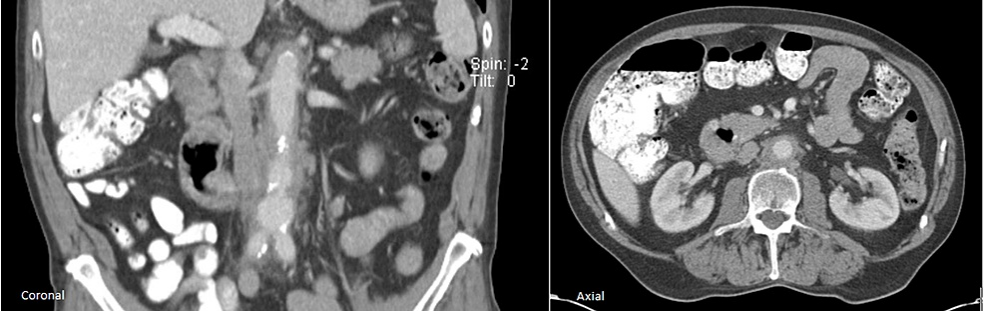
Initial CT abdomen Coronal and axial planes demonstrate a moderate degree of circumferential thickening of the periaortic space including the aortic wall. This inflammation started at the inferior thoracic aorta and extended past the aortic bifurcation till the middle of the right iliac artery CT: computed tomography

Approximately one month after the initial presentation, the patient was sent for a CT scan of his abdomen and pelvis (Figure 1), which revealed aortitis and retroperitoneal fibrosis. An inflammatory aortic aneurysm was seen with a thick peel and no saccular component. Follow-up labs shortly after his CT showed negative rheumatoid factor (RF), negative antinuclear antibodies (ANA), decrease in CRP to 10 mg/L, ESR to 27 mm/h, and ferritin to 425 ng/mL. The patient was subsequently evaluated by Vascular Surgery, at which time the patient reported no further weight loss and improvement of malaise and abdominal pain. At that time, high-dose prednisone (60 mg/day) therapy was initiated with a planned CT angiogram (CTA) in three weeks and a referral to Rheumatology.

**Figure 2:**
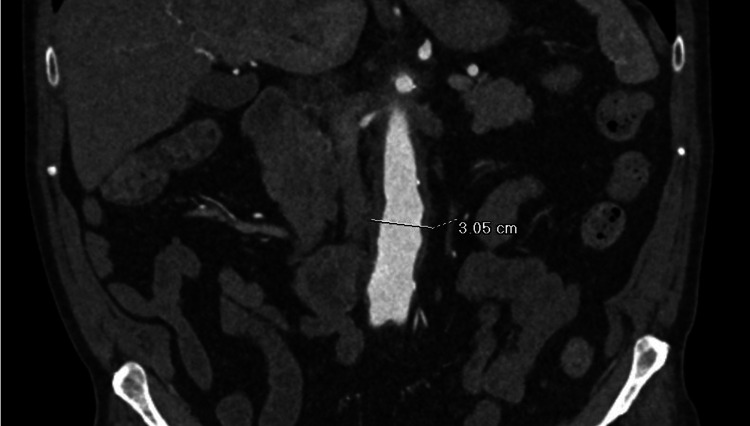
Follow-up CTA five weeks after therapy initiation Coronal image of follow-up CTA five weeks after corticosteroid therapy was initiated showed mild interval improvement in circumferential aortic inflammation. Subsequently, the proximal extent of the aortitis was found to have receded to the start of the abdominal aorta and the thoracic aortic inflammation was resolved CTA: computed tomography angiogram

**Figure 3:**
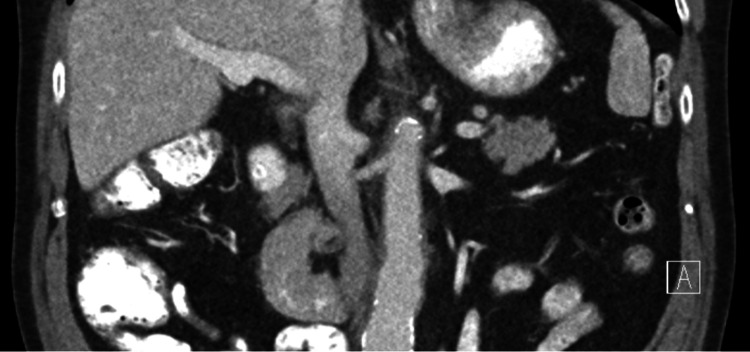
Follow-up CT eight months after therapy initiation Coronal image of follow-up CT eight months after initiation of treatment shows marked interval improvement in the circumferential inflammatory periaortic rind demonstrating improving aortitis CT: computed tomography

After the initiation of steroids, the patient’s symptoms continued to improve. CTA (Figure 2) showed slight interval improvement in the aortic/periaortic inflammation and retroperitoneal fibrosis. Follow-up labs showed an angiotensin-converting enzyme (ACE) level of 24 nmol/mL/min, negative hepatitis B/C, negative tuberculosis, negative rapid plasma reagin (RPR), and normal ESR. However, CRP had increased to 59 mg/L and ferritin to 1701 ng/mL. Because the patient felt significantly better, a steroid taper schedule was initiated, and a repeat CT (Figure 3) showed improvement.

## Discussion

Aortitis is a condition in which the layers of the aortic wall become inflamed due to a myriad of etiologies. This inflammation weakens the integrity of the vessel wall and increases the risk for aneurysm, dissection, and rupture, leading to subsequent ischemic compromise of major organs [1]. The clinical presentation of aortitis is vague and variable. Symptoms can range from fever with back or abdominal pain to severe aortic insufficiency or rupture of a thoracic abdominal aneurysm [1]. The location of the aortic inflammation or any coexisting arteritis can also impact the clinical presentation [1,2]. When aortitis is suspected, imaging is critical to establish the diagnosis. CTA is the ideal imaging technique as it allows the rapid exclusion of aortic pathologies that may mimic acute aortitis, including aortic dissection, intramural hematoma, and penetrating atherosclerotic ulcer. CTA also enables the assessment of stenotic lesions of the aorta or large arteries [1]. Modern imaging tools for the aorta also include magnetic resonance angiography (MRA) and ultrasonography [1,3].

Although the diagnosis of aortitis generally is based on clinical presentation and aortic imaging, laboratory tests are also helpful. The initial evaluation should include complete blood count (CBC), ESR, CRP, as well as kidney and liver function tests. Blood cultures should be drawn to rule out infectious causes [1]. If aortitis and retroperitoneal fibrosis are suspected, secondary causes such as lymphoma should be considered. Biopsies can confirm suspected malignancies [1].

The causes of aortitis can be broadly categorized into infectious and noninfectious. While most cases are noninfectious, it is critical to rule out infectious causes for proper treatment [1-9]. Causative organisms include *Salmonella*, *Streptococcus*, *Staphylococcus*, syphilis and tuberculosis bacteria, and viruses [1]. Most cases are bacterial with *Salmonella, Streptococcus, and Staphylococcus* being the most common causative organisms in the developed world and are associated with intravascular interventions and intravenous drug use (IVDU) [1,6]. Our patient denied any history of IVDU or high-risk sexual behavior. He did not have IVDU-associated physical exam findings, such as antecubital scarring, abscesses, or puncture lesions in different stages of healing. He also did not have physical exam findings associated with any stage of syphilis. Lab data did not show a leukocytosis, which would have been expected in a bacterial infection. A viral cause would have had a preceding prodrome, which our patient denied. Furthermore, CTA did not show the characteristic features of infectious aortitis, which include a saccular outpouching configuration and a fusiform pattern [3.6]. The lack of history, and physical, laboratory, and imaging findings ruled out an infectious cause of aortitis [2,3].

Noninfectious causes of aortic inflammation are more common and include trauma, malignancy, rheumatological connective tissue disorders, and idiopathic forms [1]. The patient had not experienced any preceding trauma. His consistently normal WBC count and lack of evidence of masses on imaging made lymphoma or other malignancy unlikely. Because our patient already had hypothyroidism, he was at an increased risk of other autoimmune disorders, which made us investigate large vessel vasculitides, such as giant cell arteritis (GCA) or Takayasu’s disease [3,7,10]. Of the two vasculitides mentioned, GCA would be the most likely condition in this patient [11]. He was over 50 years in age and had a four-day fever and nonspecific elevation of CRP and ESR [1,12,13]. However, he did not report headache, claudication of the jaw or tongue upon swallowing, visual changes, tenderness in the temporal area, or anemia, some of which are required by the diagnostic criteria set by the American College of Rheumatology (ACR) [9,10]. Given the demographics of our patient, Takayasu’s disease would be unlikely since the average age of the patients at diagnosis is 25-30 years and 75-97% of the cases are females [7,8,13]. He had no signs of limb claudication, decreased peripheral pulses, or differences in blood pressures across the limbs [8,13]. Additionally, CTA did not reveal any wall thickening of the subclavian arteries [8]. Therefore, the patient did not meet the criteria for Takayasu’s disease either [13].

Other rheumatologic disorders, including rheumatoid arthritis (RA), systemic lupus erythematosus (SLE), granulomatosis with polyangiitis, microscopic polyangiitis, and Behçet’s disease may also lead to aortitis [12-19]. In RA-associated aortitis, rheumatoid nodules are in the aortic wall in up to 50% of pathological specimens [12]. Given the lack of joint involvement and lack of RF positivity, our patient did not meet the criteria for the diagnosis of RA [20]. Cardiovascular manifestations in patients with SLE are common, but aortic aneurysm formation is rare [14]. Although our patient did present with a fever, he lacked any of the other associated symptoms such as joint pain, rash, oral ulcers as well as positive antibodies on the lupus panel. In light of this, our patient failed to meet the diagnostic criteria for SLE as well [20]. Microscopic polyangiitis and granulomatosis with polyangiitis are large vessel diseases associated with anti-neutrophil cytoplasmic antibodies (ANCA). They may present with stenosed large vessel arteritis, aneurysmal disease, aortic dissection, aortic rupture, and aortic regurgitation [15]. Our patient had none of these symptoms and again did not meet the diagnostic criteria, excluding both of these pathologies. Behçet’s disease is not commonly associated with aortitis but a few rare cases have been reported. Because there are no pathognomonic laboratory tests for Behçet’s disease, the diagnosis is made clinically and based on the absence of other systemic diseases. Patients with Behçet’s disease present with recurrent genital ulcers, eye lesions, skin lesions, and a positive pathergy test, none of which were found in our patient [16]. Since he had no associated symptoms and did not meet the diagnostic criteria, it is highly unlikely that the patient would have aortitis as a complication of a rheumatologic disorder.

An autoimmune immunoglobulin G4 (IgG4) subtype-related aortitis has been described in the literature, which can cause fibroinflammatory lesions in nearly any organ [5]. It is characterized by the thickening of the aortic wall, a lymphoplasmacytic infiltrate enriched in IgG4-positive plasma cells, and a variable degree of periaortic fibrosis [4]. According to the ACR and European League Against Rheumatism (EULAR), there is a long list of exclusion criteria as well as inclusion criteria. As per the exclusion criteria, an elevation of CRP would exclude our patient. If IgG4-related aortitis were still suspected, a biopsy would be needed for the diagnosis [4].

## Conclusions

Based on the exclusion of the aforementioned diseases and pathologies, we believe that this patient’s aortitis is idiopathic in nature. For suspected idiopathic autoimmune disease, glucocorticoids are the first-line treatment unless a direct contraindication exists. Our patient had significant symptom improvement on glucocorticoid therapy. To monitor the resolution or progression of idiopathic aortitis, we plan to follow up on inflammatory markers and imaging in this patient on a regular basis.
